# High-throughput Sequencing Reveals that BCR and TCR Repertoires as Potential Prognostic Biomarkers for Pediatric Patients with B-ALL

**DOI:** 10.2174/0113892029319425240813074610

**Published:** 2024-08-21

**Authors:** Fu Li, Xiaomei Yang, Xiuxiu Wang, Jiajia Mi, Xiao Mou, Li Song, Libo Zheng

**Affiliations:** 1 Department of Hematology and Oncology, Children's Hospital Affiliated to Shandong University and Jinan Children's Hospital, Jinan, Shandong, 250022, P.R. China;; 2 Chigene (Beijing) Translational Medical Research Center Co., Ltd., Beijing, China

**Keywords:** B-ALL, BCR/TCR repertoires, high-throughput sequencing, clinical features, biomarker, hematologic

## Abstract

**Background:**

B-ALL is a hematologic malignancy that recurs in approximately 10-20% of children and has a poor prognosis. New prognostic biomarkers are needed to improve individualized therapy and achieve better clinical outcomes.

**Methods:**

In this study, high-throughput sequencing technology was used to detect the BCR and TCR repertoires in children with B-ALL.

**Results:**

We observed a gradual increase in the diversity of the BCR and TCR repertoires during the developmental stages (Pro-, Common-, Pre-B-ALL) of precursor B-ALL cells. Conversely, as minimal residual disease (MRD) levels on day 19 of induction therapy increased, the BCR/TCR repertoire diversity decreased. Furthermore, the BCR/TCR repertoire diversity was significantly greater in B-ALL patients at low risk and those harboring the ETV6/RUNX1 fusion than in patients with medium-risk disease and those harboring the ZNF384 fusion. Notably, the abundance of BCR/TCR clones varied significantly among patients with B-ALL and different clinical characteristics. Specifically, patients with Pre-B-ALL, low-risk disease, D19MRD levels <1%, and harboring the ETV6/RUNX1 fusion exhibited a predominance of BCR/TCR small clones. In our study, we noted an imbalanced occurrence of V and J gene utilization among patients with B-ALL; however, there seemed to be no discernible correlation with the clinical attributes.

**Conclusion:**

BCR/TCR repertoires are expected to be potential prognostic biomarkers for patients with B-ALL.

## 1 INTRODUCTION

B-ALL is a clonal hematopoietic disorder characterized by the abnormal proliferation and accumulation of B-lymphoid progenitor cells [1, 2]. The treatment success rate for Acute Lymphoblastic Leukemia (ALL) has steadily increased since the 1960s, with a five-year event-free survival rate of nearly 80% for children with ALL [3-8]. Despite the progress in this field, approximately 10-20% of children will experience relapse, which is linked to an unfavorable prognosis [9-11]. Resistance and subsequent relapse following standard chemotherapy, targeted therapies, and immunotherapeutic approaches [12, 13] remain major factors contributing to pediatric cancer-related mortality. Better tools are needed to enhance treatment stratification and prognosis and to avoid overtreatment and adverse long-term side effects [14-16]. The mortality of ALL in Europe, the United States, and Japan has slightly decreased due to improvements in the treatment and development of new technologies [17]. The identification of new biomarkers of acute lymphoblastic leukemia, and thus a better understanding of their molecular basis, may lead to better monitoring of the course of the disease. Therefore, new prognostic biomarkers and more accurate risk stratifications are urgently needed to further improve individualized treatment approaches and achieve better clinical outcomes.

The T-cell receptor (TCR) and B-cell receptor (BCR) play crucial roles in T cells and B cells, respectively, enabling them to recognize antigens and participate in immune responses. T lymphocytes can be structurally classified into two subtypes, αβ and γδ, on the basis of heterodimers of T- cell receptors on their cell surface [18]. αβ T cells are found mainly in peripheral tissues and the circulatory system, while γδ T cells are found in the blood, lymphoid tissue, and epithelial environments [19]. The BCR is made up of two heavy chains (IGH) and two light chains (IGK, IGL). The genes that code for the BCR and TCR consist of variable (V), diversity (D), joining (J), and constant gene segments. The production of each BCR and TCR involves the rearrangement of multiple gene segments [20]. CDR3, or complementary determining region 3, is a short region that includes the VD and VJ junction. CDR3 is highly variable and crucial for antigen recognition [21].

Clonal rearrangements of BCRs/TCRs can be found in more than 90% of ALL patients and serve as “DNA fingerprints” for specific ALL clones. They are frequently used as sensitive targets for minimal residual disease (MRD) monitoring [22-26]. Previous studies reported that a diverse TCR repertoire in the blood is associated with a better prognosis in patients with CRC [27]. A similar association has been previously reported in tumors other than CRC, including breast [28], ovarian [29], esophageal squamous cell carcinoma [30], head and neck squamous cell carcinoma [31], and lung [32] or gastric cancer [33], among others. However, the associations between BCR/TCR clones and the clinical features of B-ALL, as well as the potential of the BCR/TCR library as a prognostic marker, remain unexplored. In this study, we characterized the TCR and BCR repertoires by detecting and analyzing CDR3 sequences in the TCR α, β, γ, and δ chains, as well as B-cell immunoglobulin (Ig) heavy (IGH) and light (IGK/IGL) chains, in B-ALL patients with different clinical characteristics. These characteristics included diverse immunophenotypes, distinct risk stratifications, varying MRD levels at day 19, and different fusion genes. Our findings provide insights into immune receptor repertoires and T-/B-cell functions in B-ALL, aiming to establish a correlation between BCR/TCR repertoires at diagnosis and prognosis in patients with B-cell lymphoblastic leukemia (B-ALL).

## 2 MATERIALS AND METHODS

### 2.1 Patients

In this study, bone marrow samples were collected from 40 newly diagnosed children with B-ALL who were admitted to the Children's Hospital of Shandong University between 2021 and 2022. Consent was acquired from the patients or their guardians before the collection of samples.

### 2.2 Sample Collection and RNA Extraction

Bone marrow samples were collected from each patient and stored in tubes containing RNAstore reagent (BioTeke, Beijing, China). Total RNA was extracted from monocytes from bone marrow samples using a blood RNA column medium extraction kit (ComWin Biotech, Beijing, China) according to the protocol recommended by the manufacturer. The quality and quantity of the extracted RNA were assessed using an Agilent 2100 Bioanalyzer (Agilent Technologies, Palo Alto, CA, USA), NanoDrop (Thermo Fisher, USA), and 1% agarose gel. Only samples with a total RNA amount of 2 µg and an RNA integrity number (RIN) ≥ 7 were used for subsequent RNA sample preparation.

### 2.3 BCR/TCR Library Construction

For BCR/TCR library construction, cDNA was generated using the 5' RACE cDNA amplification method [34]. Polymerase Chain Reaction (PCR) was then performed using specific primers targeting the complementary determining region 3 (CDR3) of the BCR/TCR to amplify the desired sequences. The purified PCR products were subjected to a second round of amplification to introduce adaptor sequences compatible with sequencing. The final libraries were assessed for quality using a Qubit dsDNA HS assay kit (Invitrogen, Carlsbad, CA, USA) on a Qubit 2.0 fluorometer.

### 2.4 mRNA Library Construction

The mRNA libraries were prepared using the mRNA-seq Lib prep kit for Illumina (AB clonal, Woburn, MA, USA) according to the protocols recommended by the manufacturer. Following library preparation, the quality of the libraries was assessed using the Qubit dsDNA HS assay kit (Invitrogen, Carlsbad, CA, USA) on a Qubit 2.0 fluorometer.

### 2.5 Sequencing

High-throughput sequencing was performed with a DNBSEQ-T7 sequencer according to the manufacturer’s instructions (BGI, Shenzhen, China). Sequencing was conducted by Beijing Chigene Translational Medicine Research Center Co., Ltd.

### 2.6 BCR/TCR-seq Quality Control and Clone Sequence Analysis

The raw sequencing data were preprocessed for adapter removal, and low-quality reads were filtered using ReSeqTools (v0.25) and FastP (v0.20.1). The original sequencing reads were aligned and assembled, and BCR/TCR sequences were obtained using MiXCR (v3.0.3). Subsequent clone sequences were analyzed, which involved annotating sequence features, comparing samples, constructing expression profiles of immune repertoires, and performing clustering analysis. To accomplish this goal, VDJtools (v1.1.4) was employed.

### 2.7 Diversity Analysis

The Chao1 index for each sample was calculated using estimate R (from the vegan package).

### 2.8 Fusion Gene Detection

The raw data were processed using fastq to remove adapters and filter out low-quality reads. The fusion genes were detected using STAR-Fusion and FusionCatcher.

### 2.9 Statistical Analysis

Statistical analysis was conducted using GraphPad Prism version 8.0 (GraphPad Software, Inc., San Diego, CA, USA). Differences among multiple groups were assessed by one-way ANOVA, followed by post hoc analyses using the Bonferroni multiple comparisons test.

## 3 RESULTS

### 3.1 Clinical Characteristics

Table **1** summarizes the specific group information for the 40 children with B-ALL who participated in the study according to different clinical characteristics. The cohort consisted of 21 male and 19 female individuals, and the treatment approach applied to most of these patients adhered to the Chinese Children's Cancer Group study ALL-2020 (CCCG-2020) protocol (Supplementary Table **1**).

### 3.2 Correlations between BCR/TCR Repertoire Diversity and Different Clinical Features in B-ALL Patients

**Fig. (1):**
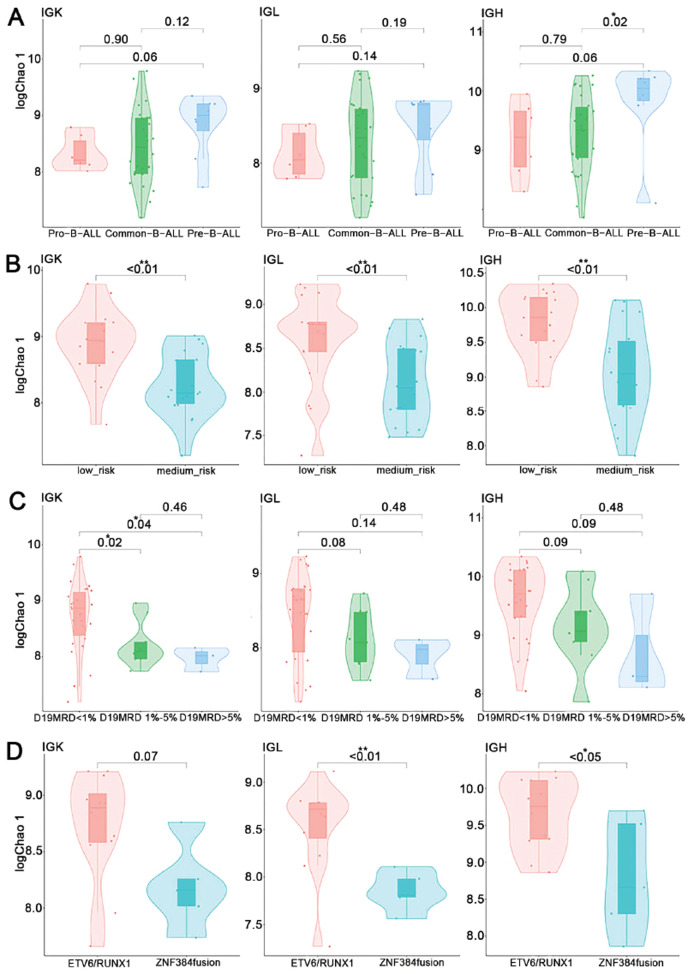
Diversity of the BCR repertoires in patients with B-ALL with different clinical features, including (**A**) different immune types, (**B**) different risk stratification, (**C**) different MRD levels on day 19, and (**D**) carrying different fusion genes. IGK/L immunoglobulin light chain, IGH immunoglobulin heavy chain. One asterisk (*) indicates *P* < 0.05, and two asterisks (**) indicate *P* < 0.01.

We conducted an analysis to examine the correlation between the richness indices (logChao1) of BCR repertoires in patients with B-ALL and various clinical features, including immune types, risk stratification, MRD levels at day 19 (D19MRD), and the presence of different fusion genes. Our findings revealed distinct patterns of BCR repertoire diversity among different immune types of B-ALL. Specifically, we observed the lowest diversity in Pro-B-ALL patients (n=6), the highest diversity in Pre-B-ALL patients (n=8), and intermediate diversity in Common-B-ALL patients (n=26) (Fig. **1A**). These results suggested that the BCR diversity in patients with B-ALL differed at different stages of B-cell differentiation, and the higher the differentiation degree of B-ALL, the greater was the BCR diversity.

We further investigated the impact of risk stratification on BCR repertoire diversity and observed that patients with low-risk B-ALL (n=17) had a significantly greater Chao 1 index compared to those with medium-risk B-ALL (n=18) (Fig. **1B**).

Additionally, we examined the association between BCR repertoire diversity and MRD levels on day 19 of induction therapy. We divided patients into three groups based on MRD on day 19 after induction therapy: those with an MRD less than 1% (n=26), those with an MRD between 1% and 5% (n=9), and those with an MRD greater than 5% (n=3). Our analysis revealed a decrease in BCR repertoire diversity as MRD levels increased (Fig. **1C**).

Moreover, we explored the potential differences in BCR repertoire diversity among patients with B-ALL and different genetic characteristics. Owing to the limited sample size, we focused on comparing patients with ETV6/RUNX1 fusion (n=12) and those with ZNF384 fusion (n=5). Our results revealed significantly greater BCR repertoire diversity in patients with ETV6/RUNX1 fusion than in those with ZNF384 fusion (Fig. **1D**).

**Fig. (2):**
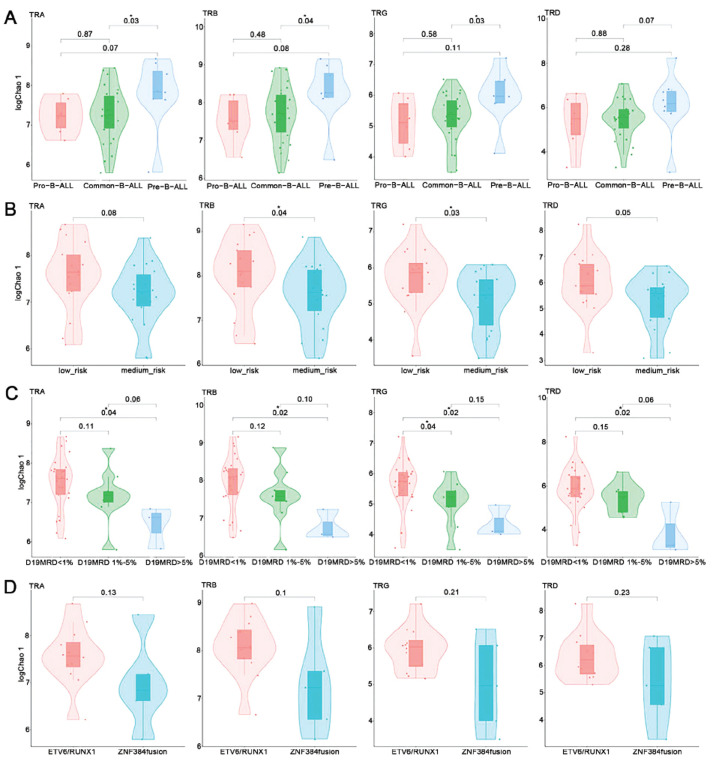
Diversity of the TCR repertoires in B-ALL patients with different clinical features, including (**A**) different immune types, (**B**) different risk stratifications, (**C**) different MRD levels on day 19, and (**D**) carrying different fusion genes. TRA T-cell receptor α, TRB T-cell receptor β, TRG T-cell receptor γ, TRD T-cell receptor δ. Asterisks (*) indicate *P* < 0.05.

We used the same method to analyze the correlation between the diversity of the TCR repertoire and various clinical features in patients with B-ALL. It was found that the correlation between TCR repertoire diversity and various clinical features in patients with B-ALL was similar to that observed for BCR. Specifically, with increasing B-cell differentiation, the TCR repertoire diversity gradually increased (Fig. **2A**). The Chao 1 index was significantly greater for low-risk compared with medium-risk patients (Fig. **2B**). Moreover, the TCR repertoire diversity was higher for patients with a low MRD burden after induction therapy (Fig. **2C**), and the TCR repertoire diversity of patients with ETV6/RUNX1 fusion was higher than of patients with ZNF384 fusion (Fig. **2D**).

These findings shed light on the dynamics of BCR/TCR repertoire diversity in patients with B-ALL and its association with clinical features, providing valuable insights into the immune landscape of this disease.

### 3.3 Association of Clonotype Abundance of BCR/TCR with Different Clinical Features in Patients with B-ALL

**Fig. (3):**
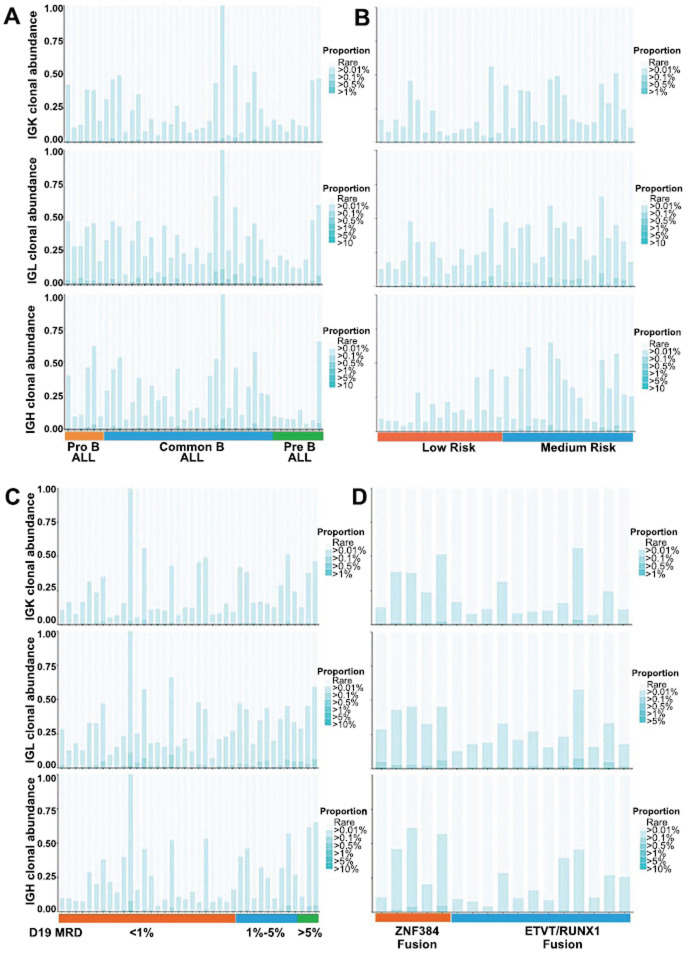
Abundances of BCR clonotypes in B-ALL patients with different clinical characteristics, including (**A**) different immune types, (**B**) different risk stratifications, (**C**) different MRD levels on day 19 after induction therapy, and (**D**) carrying different fusion genes.

Clonal size is an important feature of lymphocytes. Normally, lymphocytes are in a positive polyclonal state without any antigenic stimulation. In the case of diseases, specific antigen stimulation leads to rearrangement. Therefore, we assessed the relative abundance of BCR/TCR clonotypes by examining their frequencies within specific ranges, spanning from rare clonotypes (<0.01%) to highly abundant clonotypes (>10%). Our analysis revealed distinct patterns of clonotype distribution across different subtypes of B-ALL. Specifically, the repertoire of Pre-B-ALL patients was predominantly composed of small BCR clonotype groups, whereas highly abundant clonotypes were primarily found in Pro-B-ALL patients and common B-ALL patients (Fig. **3A**).

Furthermore, we investigated the relationship between clonotype abundance and risk stratification. It was observed that patients with low-risk B-ALL were predominantly occupied by small BCR clonotype groups, whereas highly abundant clonotypes were more prevalent in patients with medium-risk B-ALL (Fig. **3B**).

We also explored the association between clonotype abundance and minimal residual disease (MRD) levels on day 19 of induction therapy. Our results demonstrated that patients with MRD levels exceeding 1% had a relatively high abundance of BCR clonotypes (Fig. **3C**).

Furthermore, we examined the relationship between clonotype abundance and the presence of different fusion genes. Fig. (**3D**) shows that patients carrying the ETV6/RUNX1 fusion gene presented lower BCR clonal abundance than patients carrying the ZNF384 fusion gene.

**Fig. (4):**
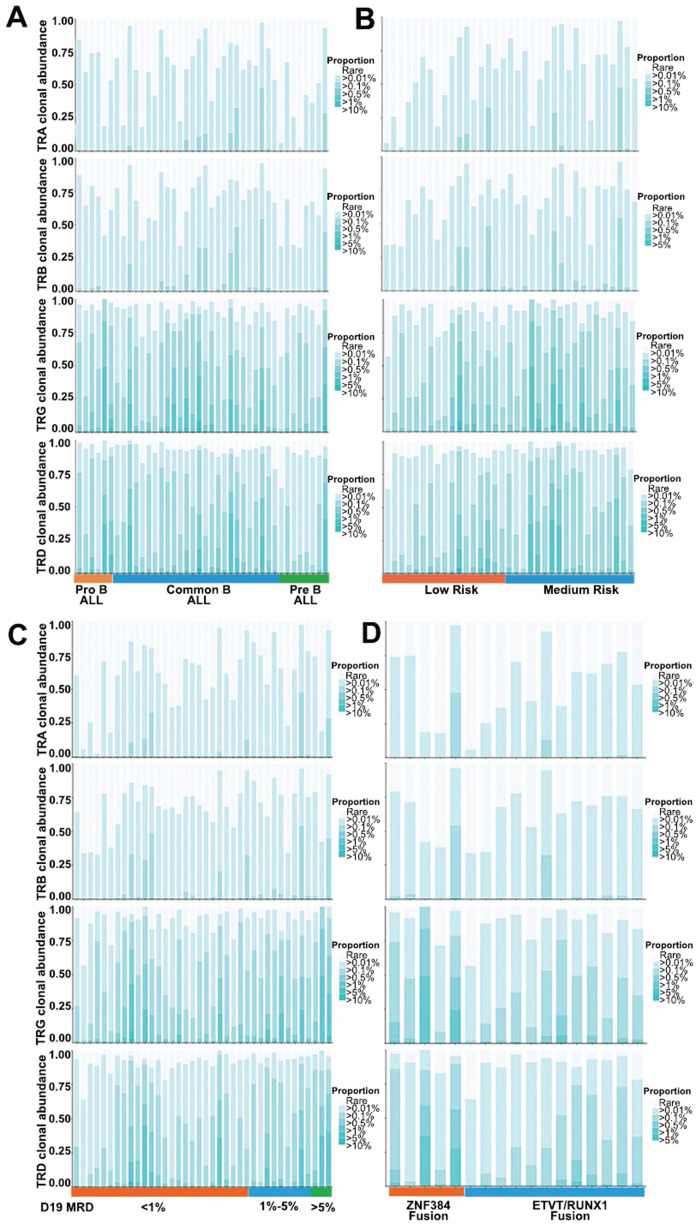
Abundances of TCR clonotypes in B-ALL patients with different clinical characteristics, including (**A**) different immune types, (**B**) different risk stratifications, (**C**) different MRD levels on day 19 after induction therapy, and (**D**) carrying different fusion genes.

We used the same method to analyze the correlation between the clonotype abundance of the TCR repertoire and various clinical features in B-ALL patients. We found that the correlation between the TCR repertoire clonotype abundance and various clinical features in B-ALL patients was similar to that in BCR patients. Specifically, the repertoire of pre-B-ALL patients was predominantly composed of small TCR clonotype groups, especially TRA chains (Fig. **4A**). As we expected, B-ALL patients with low-risk characteristics (Fig. **4B**), with MRD levels exceeding 1% (Fig. **4C**) and with ETV6/RUNX1 fusion (Fig. **4D**), were predominantly occupied by smaller TCR clonotype groups.

These findings provide insights into clonotype abundance and its association with clinical features, including subtype classification, risk stratification, MRD levels, and fusion genes, in B-ALL patients.

### 3.4 The Usage Frequencies of the V and J Genes in the BCR/TCR Repertoires of B-ALL Patients with Different Clinical Characteristics

**Fig. (5):**
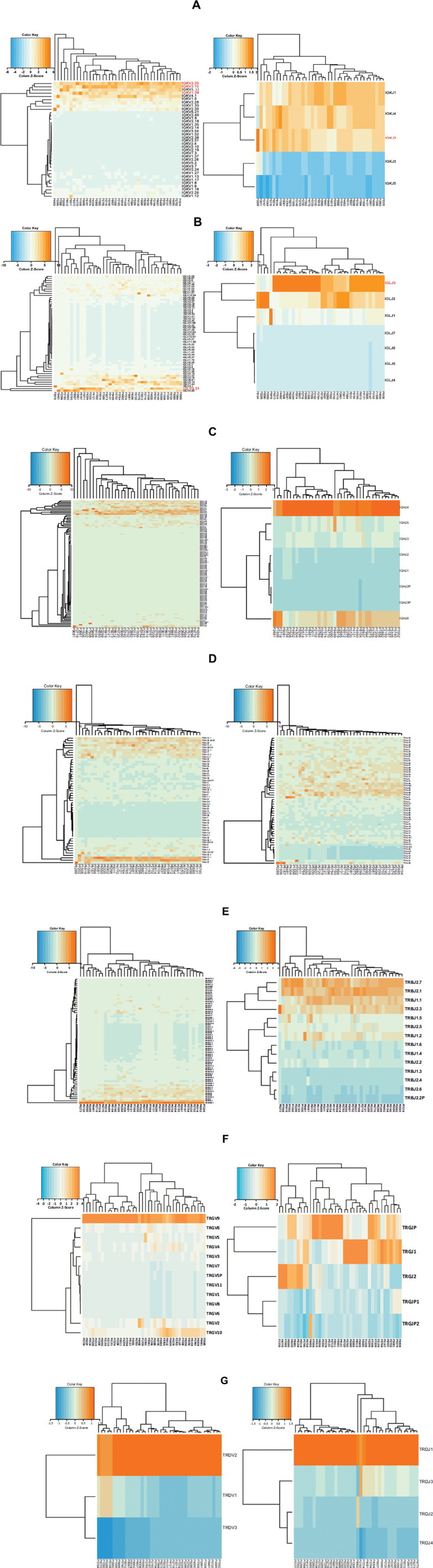
Frequencies of V and J genes in the BCR/TCR repertoires of patients with B-ALL. (**A**) Frequency of use of V and J genes in IGK, (**B**) frequency of use of V and J genes in IGL, (**C**) frequency of use of V and J genes in IGH, (**D**) frequency of use of V and J genes in TRA, (**E**) frequency of use of V and J genes in TRB, (**F**) frequency of use of V and J genes in TRG, (**G**) frequency of use of V and J genes in TRD.

We visualized the usage frequencies of the BCR/TCR V and J genes in B-ALL patients with different clinical characteristics *via* heatmaps (Fig. **5**). It was found that several V genes, including IGKV3.20 (frequency = 0.155 ± 0.033) and IGKV3.15 (frequency = 0.151 ± 0.041), were highly prevalent in nearly all the samples (Fig. **5A**). Additionally, IGKV1.39 (frequency = 0.112 ± 0.037) (Fig. **5A**) and IGLV3.21 (frequency = 0.114 ± 0.084) (Fig. **5B**) were frequently utilized in the majority of patients. The most commonly used J genes were IGKJ2 (frequency = 0.268 ± 0.030) (Fig. **5A**), IGLJ3 (frequency = 0.491 ± 0.167) (Fig. **5B**), and IGHJ4 (frequency = 0.511 ± 0.081) (Fig. **5C**).

We also examined the usage frequencies of the TCR V and J genes in B-ALL patients with different clinical characteristics. Consistent with previous reports by other researchers [35], we observed that certain TCR V genes exhibited high-frequency usage across the samples, including TRA V13.1 (frequency = 0.100 ± 0.035), TRAV21 (frequency = 0.095 ± 0.029) (Fig. **5D**), TRB V20.1 (frequency = 0.214 ± 0.091) (Fig. **5E**), TRGV9 (frequency = 0.546 ± 0.176) (Fig. **5F**), and TRDV2 (frequency = 0.822 ± 0.143) (Fig. **5G**). Similarly, TRBJ2.7 (frequency = 0.169 ± 0.037), TRBJ2.1 (frequency = 0.166 ± 0.033) (Fig. **5E**), and TRDJ1 (frequency = 0.774 ± 0.101) (Fig. **5G**) showed high-frequency usage in all samples. These findings indicate that there are skewed patterns of TCR V and J gene usage in B-ALL patients.

Finally, we observed that the use of the V and J genes in the BCR/TCR repertoire did not significantly influence the clinical characteristics of patients with B-ALL.

## 4 DISCUSSION

Genetic and epigenetic abnormalities contribute to increased proliferation potential in leukemic cells, leading to treatment resistance and eventually resulting in relapse or treatment failure [34-36]. In the last two decades, significant genomic discoveries in ALL have emerged, including RNA sequencing (RNA-seq), gene-targeting sequencing, and whole-exome sequencing (WES) [37]. These advancements have proven crucial in risk stratification, offering therapeutic insights, and bearing prognostic implications [37-39]. However, the correlation between BCR/TCR clones and the clinical characteristics of B-ALL, along with the potential of the BCR/TCR library as a prognostic marker, remains largely unexplored.

B-ALL can be categorized into different subtypes on the basis of its developmental stage, as indicated by immunophenotyping [40]. Specifically, B-ALL is classified into pro-B-ALL (CD10-), common-B ALL (CD10+), and pre-B-ALL (cytoplasmic IgM+), each with distinct characteristics. Notably, research has shown that B-ALL patients with CD10-positive expression tend to have a favorable prognosis [41], suggesting that individuals with common B-ALL might have better outcomes than those with pro-B-ALL; as expected, BCR/TCR diversity was greater in common B-ALL patients than in pro-B-ALL patients, suggesting a better prognosis with higher BCR/TCR diversity and lower clonal abundance. This finding was confirmed by subsequent results, which revealed that the BCR/TCR diversity was significantly greater and that the BCR/TCR clonal abundance was lower in low-risk patients than in intermediate-risk patients.

Additional studies have demonstrated that a high burden of MRD following induction therapy is generally associated with an unfavorable prognosis [42-44], and several additional prospective and nonrandomized studies have confirmed that MRD has a strong and independent effect on prognosis after induction and early consolidation of ALL in children and adults [45-50]. Additionally, ETV6/RUNX1 fusion is the most prevalent genetic abnormality in childhood ALL, accounting for approximately 25% of precursor-B phenotype cases [51]. Patients with this genetic rearrangement have been previously identified as a subgroup with a favorable prognosis [52-54]. In pediatric precursor B-ALL, the frequency of ZNF384 gene rearrangement is approximately 3% [55], and its prognostic impact is considered intermediate but can vary depending on the partner genes involved. This evidence suggests that patients with a low MRD load after induction therapy have a better prognosis and that patients with ETV6/RUNX1 fusion have a better prognosis than those with ZNF384 fusion. In our study, we observed that patients with low MRD loads (D19MRD levels less than 1%) after induction therapy and those with ETV6/RUNX1 fusions presented greater BCR/TCR clonal diversity and lower clonal abundance. On the basis of these observations, we speculate that patients with higher clonal diversity and lower clonal abundance might have a better prognosis. Nonetheless, owing to the relatively brief duration of the follow-up period, there are presently no statistically notable variances in the clinical results observed among the patients.

In a different context, chronic lymphocytic leukemia (CLL) with a stereotyped B-cell receptor (BCR) belonging to subset #1 (IGHV1-5-7/IGKV1-39) is known to be associated with a poor outcome [56]. Recently, the IGLV3.21 gene has emerged as a separate prognostic indicator associated with unfavorable consequences in CLL, regardless of its pairing with a heavy-chain counterpart [57-59]. We explored whether the BCR/TCR V gene or J gene in B ALL can serve as a prognostic indicator. The results revealed that TRAV13.1, TRAV21, TRBV20.1, TRGV9, TRDV2, TRBJ2.7, TRBJ2.1, and TRDJ1 exhibited high-frequency usage in almost all the samples. In these patients, skewed usage of the V and J genes of the BCR/TCR was frequently observed, as depicted by the high occurrence of IGKV3.20, IGKV3.15, IGKV1.39, IGLV3.21, IGKJ2, IGLJ3, and IGHJ4. This highlights a similar trend observed in the majority of patients. Nonetheless, we observed no significant differences in the frequency of V or J gene usage among patients with different clinical characteristics. As a result, further investigation is needed to determine whether these high-frequency V and J gene fragments can indeed serve as reliable indicators of B ALL prognosis.

The primary limitations of this study are the comparatively small sample size and the relatively brief duration of the follow-up period. With a larger study group and longer follow-up time, we could have conducted more sophisticated analyses specifically aimed at identifying BCR/TCR differences among patients with B-ALL who exhibit diverse clinical characteristics. This could have facilitated a more in-depth exploration of novel prognostic markers, ultimately leading to the advancement of individualized therapy and the achievement of better clinical outcomes.

## CONCLUSION

In conclusion, our comprehensive analysis of the TCR and BCR repertoires of B-ALL patients with different clinical characteristics highlights their potential as prognostic biomarkers. Incorporating BCR/TCR repertoire analysis at the time of diagnosis can serve multiple purposes. It can provide baseline data for monitoring MRD [60], and additionally, it offers valuable insights into potential prognostic indicators, aiding in the formulation of personalized treatment strategies to improve patient outcomes.

## HIGHLIGHTS

1. Our findings shed light on the dynamics of BCR/TCR repertoire diversity in B-ALL patients and its associations with clinical features.
2. The abundance of BCR/TCR clones varied significantly among B-ALL patients with different clinical characteristics.
3. The use of V and J genes in the BCR/TCR repertoire did not significantly influence the clinical characteristics of patients with B-ALL.
4. The BCR/TCR repertoire is expected to be a potential prognostic biomarker for patients with B-ALL.

## AUTHORS' CONTRIBUTIONS

Fu Li and Xiaomei Yang developed the concept and design of the study. Xiao Mou, Li Song, and Libo Zheng collected samples and participated in the experiments. Jiajia Mi collected and analyzed the data. Xiuxiu Wang participated in the experiments, interpreted the data, and conceptualized and wrote the manuscript. Fu Li provided valuable input into the study and reviewed and approved the manuscript. All of the authors critically evaluated the manuscript and approved its submission.

## Figures and Tables

**Table 1:** Specific groups according to different clinical characteristics.

**Samples**	**EGIL-1995**	**Samples**	**Risk Stratification**	**Samples**	**D19 MRD**	**Samples**	**Fusion Gene**
P2702	Pro B ALL	P2765	Low risk	P9081	<0.01%	P7574	EWSR1/ZNF384
P9081	Pro B ALL	P7209	Low risk	P2765	<0.01%	P7994	TCF3/ZNF384
P9273	Pro B ALL	P7574	Low risk	P7574	<0.01%	P0289	TAF15/ZNF384; ZNF384/TAF15
P7994	Pro B ALL	P0131	Low risk	P8512	<0.01%	P9273	TCF3/ZNF384
P0289	Pro B ALL	P4550	Low risk	P4550	<0.01%	P7508	EP300/ZNF384; ZNF384/EP300
P4902	Pro B ALL	P1506	Low risk	P1506	<0.01%	P0754	RUNX1/ETV6; ETV6/RUNX1
P2765	Common B ALL	P4121	Low risk	P0514	<0.01%	P2765	RUNX1/ETV6; ETV6/RUNX1
P2685	Common B ALL	P5167	Low risk	P2193	<0.01%	P4121	ETV6/RUNX1
P4304	Common B ALL	P0514	Low risk	P3011	<0.01%	P5167	ETV6/RUNX1
P7209	Common B ALL	P3011	Low risk	P2882	<0.01%	P2719	ETV6/RUNX1
P7574	Common B ALL	P2882	Low risk	P1556	<0.01%	P0131	ETV6/RUNX1; RUNX1/ETV6
P8512	Common B ALL	P5335	Low risk	P2719	<0.01%	P0514	ETV6/RUNX1; RUNX1/ETV6
P0131	Common B ALL	P2719	Low risk	P0754	<0.01%	P5335	ETV6/RUNX1; RUNX1/ETV6
P0720	Common B ALL	P0754	Low risk	P8104	<0.01%	P8481	ETV6/RUNX1; RUNX1/ETV6
P4550	Common B ALL	P8104	Low risk	P2661	0.01%	P1556	ETV6/RUNX1; RUNX1/ETV6
P4770	Common B ALL	P2661	Low risk	P5167	0.04%	P2661	ETV6/RUNX1; RUNX1/ETV6
P5740	Common B ALL	P0090	Low risk	P5335	0.05%	P2882	ETV6/RUNX1; RUNX1/ETV6; TBC1D15/RAB21
P5919	Common B ALL	P2702	Medium risk	P2299	0.09%	P5740	PAX5/JAK2
P8937	Common B ALL	P9081	Medium risk	P4121	0.12%	P9499	TCF3/PBX1
P1506	Common B ALL	P7994	Medium risk	P9499	0.14%	P4902	AFF1/KMT2A; KMT2A/AFF1
P4121	Common B ALL	P0289	Medium risk	P0090	0.21%	P2702	NA
P5167	Common B ALL	P4902	Medium risk	P8937	0.31%	P9081	NA
P0514	Common B ALL	P4304	Medium risk	P8296	0.35%	P2685	NA
P2299	Common B ALL	P8512	Medium risk	P7209	0.36%	P4304	NA
P2193	Common B ALL	P0720	Medium risk	P0131	0.50%	P7209	NA
P3011	Common B ALL	P4770	Medium risk	P4304	0.69%	P8512	NA
P2882	Common B ALL	P5740	Medium risk	P4902	1.30%	P0720	NA
P5335	Common B ALL	P8937	Medium risk	P7002	2%	P4550	NA
P7002	Common B ALL	P2299	Medium risk	P5740	2.19%	P4770	NA
P7508	Common B ALL	P7002	Medium risk	P2702	2.36%	P5919	NA
P8481	Common B ALL	P7508	Medium risk	P4770	2.95%	P8937	NA
P1556	Common B ALL	P8481	Medium risk	P7994	3.63%	P1506	NA
P2719	Pre B ALL	P1556	Medium risk	P7508	3.78%	P2299	NA
P9499	Pre B ALL	P9499	Medium risk	P8481	3.87%	P2193	NA
P0754	Pre B ALL	P3764	Medium risk	P0720	4.93%	P3011	NA
P8104	Pre B ALL	P9273	High risk	P3764	5.78%	P7002	NA
P8296	Pre B ALL	P5919	High risk	P0289	26.99%	P8104	NA
P2661	Pre B ALL	P2685	/	P9273	30.10%	P8296	NA
P0090	Pre B ALL	P2193	/	P2685	/	P0090	NA
P3764	Pre B ALL	P8296	/	P5919	/	P3764	NA

**Note:** EGIL: Leukemia immunotyping European group

NA: No gene fusion was detected

## Data Availability

The authors confirm that the data supporting the findings of this research are available within the article.

## LIST OF ABBREVIATIONS

ALL: Acute Lymphoblastic Leukemia
BCR: B-cell Receptor
CDR3: Complementary Determining Region 3
CLL: Chronic Lymphocytic Leukemia
MRD: Minimal Residual Disease
PCR: Polymerase Chain Reaction
TCR: T-cell Receptor
WES: Whole-exome Sequencing
